# COVID-19 Confinement and Sexual Activity in Spain: A Cross-Sectional Study

**DOI:** 10.3390/ijerph18052559

**Published:** 2021-03-04

**Authors:** Rubén López-Bueno, Guillermo F. López-Sánchez, Alejandro Gil-Salmerón, Igor Grabovac, Mark A. Tully, José Casaña, Lee Smith

**Affiliations:** 1 Department of Physical Medicine and Nursing, University of Zaragoza, 50009 Zaragoza, Spain; 2 Vision and Eye Research Institute, School of Medicine, Faculty of Health, Education, Medicine and Social Care, Anglia Ruskin University-Cambridge Campus, Cambridge CB1 1PT, UK; 3 Polibienestar Research Institute, University of Valencia, 46022 Valencia, Spain; alejandro.gil.salmeron@uv.es; 4 Centre for Public Health, Department of Social and Preventive Medicine, Medical University of Vienna, 1090 Vienna, Austria; igor.grabovac@meduniwien.ac.at; 5 Institute of Mental Health Sciences, School of Health Sciences, Ulster University, Newtownabbey BT37 0QB, UK; m.tully@ulster.ac.uk; 6 Exercise Intervention for Health Research Group (EXINH-RG), Department of Physiotherapy, University of Valencia, 46022 Valencia, Spain; jose.casana@uv.es; 7 The Cambridge Centre for Sport and Exercise Sciences, Anglia Ruskin University, Cambridge CB1 1PT, UK; Lee.Smith@aru.ac.uk

**Keywords:** sexual intercourse, social isolation, COVID-19, health habits, lifestyle

## Abstract

Restrictions of free movement have been proven effective in tackling the spread of COVID-19 disease. However, sensitive populations submitted to longer periods of restrictions may experience detrimental effects in significant areas of their lifestyle, such as sexual activity. This study examines sexual activity during the COVID-19 confinement in Spain. A survey distributed through an institutional social media profile served to collect data, whereas chi-squared tests, *t*-tests, analyses of variance, and multiple logistic regression analysis were used to assess differences among sample subgroups. A total of 71.3% adults (N = 536) (72.8% female) reported engaging in sexual activity with a weekly average of 2.39 times (SD = 1.80), with significant differences favoring males, middle age, married/in a domestic relationship (*p* < 0.001), employed (*p* < 0.005), medium–high annual household income, living outside the Iberian Peninsula, and smoking and alcohol consumption. Analyses adjusted for the complete set of control variables showed significant odds for a lower prevalence of weekly sexual activity in women (OR = 0.44, 95% CI 0.27–0.72). Interventions to promote sexual activity in confined Spanish adults may focus on groups with lower sexual activity.

## 1. Introduction

In March 2020, the World Health Organization (WHO) declared the COVID-19 outbreak a global pandemic [1]. COVID-19 is caused by SARS-CoV-2, a variant of coronavirus. As of 17 April 2020 (10:00 a.m. CET), more than 2,160,170 cases have been diagnosed globally, with over 68,976 fatalities. COVID-19 is a respiratory virus that is transmitted by large respiratory droplets and direct contact with infected secretions [2].

In order to tackle the spread of the virus SARS-CoV-2, the Spanish Government approved a period of confinement due to COVID-19 on 15 March 2020; during the confinement period, enacted measures comprised free movement restrictions and imposed social distancing, thus, owing to these restrictions, daily routines were likely to be dramatically affected [3]. A relevant behavior that is likely to be impacted by the Spanish social distancing guidance is sexual behavior, which can encompass a plethora of acts including penetrative sex (vaginal, anal), oral sex, and mutual masturbation, although the information on this topic among the Spanish population remains unknown.

According to the WHO, sexual health is considered a state of physical, emotional, mental, and social well-being regarding sexuality, which is linked to the possibility of having pleasurable and safe sexual experiences [4]. Importantly, a frequent and trouble-free sex life is associated with a myriad of physical and mental health benefits. For example, sexual inactivity has been found to be significantly associated with a higher risk of cancer, bladder/bowel problems, major surgery, poor vision, mental health conditions, and cardiovascular disease and its risk factors, including diabetes, hypertension, and high cholesterol [5]. A higher frequency of sexual activity has been shown to be beneficial for mental health since frequent sexual activity is associated with greater enjoyment of life [6], quality of life [7], well-being [8], and cognitive function [9] in older adults. Moreover, the benefits of regular sexual activity also extend to populations of younger adults; a recent study by Carcedo et al. [10] observed higher levels of sexual satisfaction to be associated with lower levels of depression among Spanish young adults, which in turn has been observed to be mediated by the frequency of sexual activity [11]. Despite this knowledge, trends in the frequency of sexual inactivity among adults aged 18 to 44 years have importantly increased over recent decades in countries such as the United States [12]. Similarly, a declining trend regarding the frequency of sexual activity has been recently identified in Britain, particularly more accentuated among those in early middle age and those who were married or cohabiting [13].

In a recent study carried out on a sample of 868 UK adults whilst following UK COVID-19 social distancing guidance, just 39.9% of the population reported engaging in sexual activity at least once per week [14]. To date, little is known about sexual behavior during COVID-19 social distancing. Due to social differences between countries, it is not known whether sexual activity in Spain during COVID-19 social isolation would be at a similar level and whether the same correlates exist; prior research has shown Spanish sexual activity (i.e., sexual intercourse) to be substantially higher in comparison to other countries [15]. However, the Spanish population has been experiencing stricter confinement measures and movement restrictions due to COVID-19 than populations from other countries such as the United Kingdom during consecutive months. This might have profoundly impacted their mental health, which, in turn, may have led to lower levels of sexual activity.

Therefore, the aim of the present study is to investigate levels and correlates of sexual activity during COVID-19 self-isolation/social distancing in a sample of the Spanish adult population using a cross-sectional online survey.

## 2. Materials and Methods

### 2.1. Study Design

An observational study involving a cross-sectional online survey investigating the health behaviors of adults was carried out during the COVID-19 outbreak in Spain. A publication about the survey was posted on a social media institutional account inviting adults aged 18 years or over, currently residing in Spain and confined due to COVID-19, to participate. Data collected through the survey comprised of the 22nd of March (i.e., the 8th day of enacted confinement due to COVID-19 in Spain) up to the 23rd of May (i.e., the end of the strict confinement). Convenience sampling was conducted to select the participants of the study; according to server analytics, 856 media users covering all the Spanish regions were asked to participate. Those individuals who accessed the online web link to participate in the survey were first directed to a web form (SoSci Survey, SoSci Survey GmbH, Munich, Germany), where they were required to give informed consent to participate in the survey without any payment compensation. Participants were informed that they could stop completing the survey at any point without consequences. Before completing the survey, participants were asked to confirm their confined status, their current residence in Spain, and their age. If the potential participant’s response was affirmative to all these questions, the participant was asked to complete the survey. Overall, 536 respondents (63% of the participants) with complete information regarding the examined variables were included in the study. This study adhered to the Strengthening the Reporting of Observational Studies in Epidemiology (STROBE) reporting guidelines 11. The study was conducted in accordance with the principles of the World Medical Declaration of Helsinki and approved by the Ethics Committee of Research in Humans of the University of Valencia (register code 1278789).

### 2.2. Dependent Variable

In the online survey, sexual activity was defined as masturbation, sexual intercourse, petting, or fondling. Participants were asked: “On average, while confined, how many times have you engaged in sexual activity weekly?”.

### 2.3. Independent Variables

Collected demographic data were conducted through single-item questions: “What is your gender?” and potential answers comprised “male” or “female”, “What is your age?”, and potential answers included 10-year age bands, “What is your marital status?”, with potential answers including “single”, “divorced”, “separated”, “widowed”, or “married/in a domestic partnership”, “What is your current status?”, and possibilities comprised “employed”, and “not employed”, “What is your average household annual income?”, and possible answers included “<€15,000”, “€15,000–<€25,000”, “€25,000–<€40,000”, “€40,000–<€60,000”, “≥€60,000”. Participants were also asked whether they were residing in the Iberian Peninsula or other Spanish regions outside the Iberian Peninsula (“What part of the country do you live in?”), and possible answers comprised “Peninsula”, “Balearic Islands”, “Canary Islands”, and “Ceuta or Melilla”. Measures of health status included whether respondents were a current consumer of alcohol (“Do you drink alcohol?”), and a smoker (“Do you smoke?”), and possible answers for these two questions comprised “yes”, or “no”; “Have you ever been diagnosed by a health professional with: (tick all that apply)”: “hypertension”, “obesity”, “myocardial infarction”, “angina pectoris and other coronary diseases”, “other cardiac diseases”, “varicose veins of lower extremities”, “osteoarthritis”, “chronic neck pain”, “chronic low back pain”, “chronic allergy (excluding allergic asthma)”, “asthma (including allergic asthma)”, “chronic bronchitis”, “emphysema or chronic obstructive pulmonary disease (COPD)”, “type 1 diabetes”, “type 2 diabetes”, “diabetic retinopathy”, “peptic ulcer disease”, “cataract”, “urinary incontinence or urine control problems”, “hypercholesterolemia”, “chronic skin disease”, “chronic constipation”, “liver cirrhosis and other hepatic disorders”, “stroke”, “chronic migraine and other frequent chronic headaches”, “hemorrhoids”, “cancer”, “osteoporosis”, “thyroid disease”, “injury”, and “renal disease”. Psychiatric conditions included both anxiety and depression estimated through the Spanish version of Beck inventories of depression and anxiety [12,13] as well as the option to declare “other psychiatric conditions”. Moreover, participants were asked if they had experienced any physical symptoms of COVID-19 during the confinement: “What guidelines are you following for self-isolation?”, and responses comprised the selection of one or more of the following symptoms: “high temperature”, “persistent cough”, “sore throat”, and “runny nose”. Finally, the participants were asked for the number of days they had been confined (“What day of self-isolation are you currently on?”).

### 2.4. Statistical Analyses

The statistical analysis was performed with Stata v16.1. Participants with and those without sexual activity were compared in relation to sample characteristics using chi-squared tests for categorical variables and t-tests for continuous variables. The normality of sexual activity was previously confirmed through a Kolmogorov–Smirnov test. Furthermore, the average number of sexual activities was compared between males and females, and between the different age groups (i.e., 18–24 years, 25–34 years, 35–44 years, 45–54 years, 65–74 years), using independent t-tests and analysis of variance (ANOVA), respectively. Effect sizes were estimated using a phi coefficient (chi-squared tests with binary categorical variables), Cramer’s V (chi-squared tests with categorical variables with more than 2 categories), Cohen’s d (independent t-tests with continuous variables), and eta squared (ANOVA with continuous variables). To check associations between weekly prevalence of sexual activity and sex and age subgroups, we conducted a multiple logistic regression adjusted for control variables (marital status, employment, average household annual income, place of living, current smoking, current alcohol consumption, physical condition, psychiatric condition, physical symptoms, and days of confinement). Participants with missing values were removed from the analyses, and a complete case analysis was carried out. No significant differences regarding any of the control variables were identified between those participants who responded to the questions concerning study variables and those who did not. The level of statistical significance was set at *p* < 0.05.

## 3. Results

There were 536 individuals included in this study (72.8% women, 27.0% aged 18 to 24 years (*n* = 145), 35.6% aged 25 to 34 years (*n* = 191), 26.9% aged 35 to 44 years (*n* = 144), 6.7% aged 45 to 54 years (*n* = 36), 3.2% aged 55 to 64 years (*n* = 17), and 0.6% aged 65 to 74 years (*n* = 3); Table 1). During COVID-19 confinement, 71.3% of the population (N = 382) reported engaging in sexual activity at least once per week on average and were thus classified as sexually active. There was a particularly high prevalence of sexual activity for male, middle age, married/in a domestic relationship (*p* < 0.001), employed (*p* < 0.005), medium–high annual household income, living outside the Iberian Peninsula (i.e., Balearic Islands, Canary Islands, Ceuta or Melilla), current smoking and alcohol consumption factors in adults with sexual activity compared to those without sexual activity (Table 1), while the number of chronic psychiatric conditions was significantly lower in the sexually active than in the non-sexually active group (*p* < 0.05; Table 1). The mean weekly number of sexual activities (i.e., masturbation, petting, fondling, or sexual intercourse) was 2.39 (SD = 1.80) in the overall population, and this number was slightly higher in men than in women (2.49 versus 2.36; Table 2). Furthermore, the weekly average of sexual activity steadily decreased from 2.61 (SD = 1.76) in the younger subgroup of participants to 2.33 (SD = 0.58) in the older subgroups of participants (i.e., each age subgroup showed a progressively lower weekly average of sexual activity as regards the participants aged 18 to 24 years with the exception of the subgroup of participants aged 55 to 64 years), although these values do not show significant differences among subgroups. Finally, Table 3 shows associations between the prevalence of weekly sexual activity in relation to sex and age subgroups. In the full adjusted model (Model 3), only the female subgroup showed significant odds for a lower prevalence of weekly sexual activity (OR = 0.44, 95% CI 0.27–0.72).

## 4. Discussion

For the first time, the present study describes the levels of sexual activity in a sample of Spanish adults during COVID-19 social distancing. The present study identified higher sexual activity levels than what has been observed in the UK during the COVID-19 confinement (39.9% versus 71.3%) [14]. These differences in levels of sexual activity may be due to social attitudes towards sexual activity in Spain compared to the UK, since levels of sexual activity remain particularly high among older Spanish adults, especially in males [16], even when compared with Spanish adolescents [17], Furthermore, another study observed 39.9% males and 29.9% females from a Spanish population aged 40 to 80 years to engage in regular sexual intercourse (i.e., more than once a week) in normal conditions [15], thus, confinement may not have strongly influenced the sexual activity, particularly that of those who are married or in a domestic partnership.

The findings from the present study support those from prior research relating to sexual activity and social distancing, showing that those who are male, younger or middle aged, married or in a domestic partnership, and consume alcohol are more likely to engage in sexual activity [14]. In addition, the present study also found that those living outside the Iberian Peninsula and those who smoke were also more likely to engage in sexual activity. However, since worldwide traffic regarding popular pornographic websites compared to the situation prior to COVID-19 increased during confinement, a change of sexual habits to avoid potential COVID-19 infection should not be disregarded, particularly in more vulnerable populations. Fear of infection, lack of intimacy, especially among those confined with children, and the subsequent stress generated by the situation might increase masturbation frequency that is usually linked to pornography consumption; indeed, a 6% higher pornography consumption compared with normal values was observed during the confinement in Spain [18], with similar values shown in other European countries such as France or Italy [19]. Moreover, how the confinement might have affected relationships remains unknown, although it is expected to have worsened sexual activity. For example, divorce applications increased in the most affected provinces of China, which in turn might have reduced sexual intercourse [18]. Furthermore, higher exposure to the pandemic such as that experienced by health workers and their acquaintances has been associated with significantly lower levels of sexual desire during the Italian confinement [20]. This phenomenon has been previously observed in other traumatic events such as earthquakes or hurricanes [21,22]. Interestingly, it was observed in a cross-national study with participants from several South-East Asian countries that few differences existed among participants. It is thus possible that either cultural differences or different COVID-19 implemented measures between countries might explain different findings between studies on this topic [23]. Further research of a qualitative nature is warranted to both confirm as well as better understand the reasons underlying these potential sex, age, socioeconomic, and cross-cultural differences.

In contrast to previous research underscoring the association between chronic conditions and number of sexual partners (i.e., a possible indicator of higher sexual activity) [24], the present study did not identify differences in sexual activity in relation to the number of either physical or psychiatric chronic conditions during COVID-19 confinement in Spain. This result could be partly explained by the fact that restriction of movements might have affected those participants with multiple sexual partners and this may have reduced their sexual activity. Interestingly, our results regarding alcohol consumption and sexual activity endorse those found previously by Grabovac et al. [25], as participants with current alcohol drinking consumption showed higher levels of sexual activity than their counterparts. Owing to reduced levels of physical activity during COVID-19 confinement, which might have worsened mood [26], levels of alcohol consumption along with sexual activity possibly increased over this period.

Limitations of the study include the possibility of a self-reporting bias in the findings; furthermore, how the definition of sexual activity was interpreted by the participants might lead to biased results (e.g., one participant might interpret petting and masturbation as one single sexual activity when they occurred consecutively, and another might have interpreted this as two different sexual activities). Furthermore, interpretations of the results from this study, when compared with others, should be made considering this wide sexual activity definition. Second, the analysis was cross-sectional, hence it was not possible to investigate trajectories of sexual activity during the COVID-19 pandemic in Spain; although the authors did not identify significant differences regarding sexual activity and number of isolation weeks for the participants (results not published), the influence of time in longer studies should not be ruled out. Due to their brevity, single-item questions have been recommended for use in specific contexts of illness and frailty, thus we decided to use those in this unprecedented confinement context [27]. Furthermore, due to the necessity of collecting data at the beginning of the confinement, validating questions regarding sexual activity was not feasible. Finally, due to the type of sampling, there is an overrepresentation of women and younger participants, hence the possibility of a selection bias that increased the average number of sexual activities.

## 5. Conclusions

In the present sample of social distancing Spanish adults, a high prevalence of sexual activity was observed in comparison to similar data from the UK. Interventions to promote sexual activity in social distancing Spanish adults may want to focus on females, older age, those who are not married, those who abstain from alcohol, non-smokers, and those living in the Iberian Peninsula.

## Figures and Tables

**Table 1:** Sample characteristics (overall and by sexual activity status).

Characteristics	Category	Overall (N = 536)	Sexual Activity		Effect Size ^a^	*p*-Value ^b^
			No (N = 154)	Yes (N = 382)		
Sex	Male	27.2	18.5	81.5	0.14	0.001
	Female	72.8	32.6	67.4		
Age	18–24 years	27.0	38.6	61.4	0.16	0.021
	25–34 years	35.6	23.6	76.4		
	35–44 years	26.9	24.3	75.7		
	45–54 years	6.7	27.8	72.2		
	55–64 years	3.2	35.3	64.7		
	65–74 years	0.6	66.7	33.3		
Marital status	Single/separated/divorced/widowed	66.8	34.6	65.4	0.19	<0.001
	Married/in a domestic partnership	33.2	16.9	83.1		
Employment	No	30.8	37.6	62.4	0.13	0.003
	Yes	69.2	24.8	75.2		
Annual household income	Less than EUR 15,000	52.8	32.5	67.5	0.10	0.253
	EUR 15,000 to 25,000	33.8	25.4	74.6		
	EUR 25,000 to 40,000	10.6	22.8	77.2		
	EUR 40,000 to 60,000	1.7	11.1	88.9		
	More than EUR 60,000	1.1	33.3	66.7		
Living outside the peninsula (Balearic Islands, Canary Islands, Ceuta or Melilla)	No	81.7	30.4	69.6	0.08	0.077
	Yes	18.3	21.4	78.6		
Current smoking	No	84.5	29.8	70.2	0.06	0.201
	Yes	15.5	22.9	77.1		
Current alcohol consumption	No	60.8	30.4	69.6	0.05	0.297
	Yes	39.2	26.2	73.8		
Number of chronic physical conditions		0.80 (1.26)	0.85 (1.20)	0.79 (1.29)	0.05	0.588
Number of chronic psychiatric conditions		0.13 (0.37)	0.18 (0.40)	0.10 (0.35)	0.19	0.044
Any physical symptom experienced during confinement	No	8.6	21.7	78.3	0.05	0.273
	Yes	91.4	29.4	70.6		
Number of days of confinement		29.8 (7.7)	30.3 (6.5)	29.7 (8.1)	0.08	0.421

Sexual activity was dichotomized into sexual activity (at least one sexual intercourse per week on average) versus no sexual activity (zero sexual intercourse per week on average). Values are percentages unless otherwise stated. ^a^ Effect size was calculated using phi coefficient and Cramer’s V for categorical variables and Cohen’s d for continuous variables. ^b^ *p*-values were based on chi-squared tests for categorical variables and on independent t-tests for continuous variables.

**Table 2:** Mean number of sexual activities per week in the overall population and by sex and age.

Population	Mean (Standard Deviation)	Effect Size ^a^	*p*-Value ^b^
Overall	2.39 (1.80)	-	-
Sex			
Male	2.49 (1.73)	0.07	0.470
Female	2.36 (1.82)		
Age			
18–24 years	2.61 (1.76)	0.01	0.651
25–34 years	2.36 (1.86)		
35–44 years	2.28 (1.94)		
45–54 years	2.36 (0.90)		
55–64 years	2.06 (1.60)		
65–74 years	2.33 (0.58)		

^a^ Effect size was calculated using Cohen’s d for the sex analysis and eta squared for the age analysis; ^b^ *p*-values were obtained using independent *t*-test and analysis of variance.

**Table 3:** Adjusted odds ratios (95% confidence interval) for prevalence of weekly sexual activity in relation to sex and age subgroups (references male and 18–24 years subgroups respectively).

	Model 1 ^a^ OR (95% CI)	Model 2 ^b^ OR (95% CI)	Model 3 ^c^ OR (95% CI)
Sex			
Male	1	1	1
Female	0.46 (0.28–0.73)	0.44 (0.27–0.71)	0.44 (0.27–0.72)
Age			
18–24 years	1	1	1
25–34 years	2.20 (1.36–3.56)	1.54 (0.87–2.72)	1.61 (0.90–2.89)
35–44 years	2.04 (1.22–3.40)	0.94 (0.47–1.89)	1.00 (0.49–2.08)
45–54 years	1.87 (0.83–4.21)	0.65 (0.23–1.83)	0.75 (0.26–2.18)
55–64 years	1.42 (0.49–4.09)	0.58 (0.18–1.94)	0.64 (0.18–2.28)
65–74 years	0.30 (0.03–3.53)	0.21 (0.18–2.42)	0.18 (0.01–2.14)

OR Odds Ratio; CI Confidence Interval; ^a^ Model 1 Adjusted for age (sex) or sex (age); ^b^ Model 2 Adjusted for Model 1 and socioeconomic variables (marital status, employment, annual household income, and place of living); ^c^ Model 3 Adjusted for Model 2, days of confinement, and health variables (current smoking, current alcohol consumption, psychiatric conditions, physical conditions, physical symptoms).

## Data Availability

The data that support the findings of this study are available from the corresponding authors, upon reasonable request.
